# Predictive Values of Early Parental Loss and Psychopathological Risk for Physical Problems in Early Adolescents

**DOI:** 10.3389/fpsyg.2018.00922

**Published:** 2018-06-06

**Authors:** Mimma Tafà, Luca Cerniglia, Silvia Cimino, Giulia Ballarotto, Eleonora Marzilli, Renata Tambelli

**Affiliations:** ^1^Department of Dynamic and Clinical Psychology, Sapienza Università di Roma, Rome, Italy; ^2^Faculty of Psychology, Università Telematica Internazionale Uninettuno, Rome, Italy

**Keywords:** parental loss, psychopathological risk, physical illness, hazard regression analysis, adolescence

## Abstract

**Background:** Several studies have suggested that the early loss of parents is a potentially traumatic experience, exposing adolescents to a higher risk for the onset of psychopathological symptoms. Furthermore, research has shown an association between the loss of a parent in childhood and subsequent physical illnesses, but much less attention has been given to the predictive role of loss in the development of physical illness in adolescence.

**Methods:** From a larger normative sample, we selected 418 early adolescents (and their surviving parents) each of whom had lost a parent in their first 3 years of life. We evaluate the offspring’s and parents’ psychopathological symptoms, dissociation, and physical problems over a 6-year period. Univariate and multivariate Cox proportional hazard regression analyses with time-dependent variables were used to examine the predictive values of the adolescents’ and surviving parents’ psychopathological symptoms, and youths’ demographic characteristics (sex and age) for the occurrence of physical illness during a 6-year period of follow-up.

**Results:** Independently of sex, the psychopathological risk of the surviving parents’ and adolescents’ affective problems and dissociation has been found to predict the occurrence of physical illnesses. Furthermore, dissociation was the most significant predictor of significant physical problems.

**Conclusion:** These results may be relevant and an addition to the previous literature, opening up new possibilities for prevention and intervention that are oriented toward greater support for children who have experienced the loss of one parent and for surviving parents.

## Introduction

The death of a parent in the 1st year of a child’s life represents one of the most significant painful events in his or her emotional development (Haine et al., 2008), which may have short-term (Rostila and Saarela, 2011) and/or long-term consequences for mental health (Ellis et al., 2013), as well as causing physical illness (Bylund-Grenklo et al., 2016; Cipriano and Cipriano, 2017). Although the death of a parent is a potentially traumatic event when it happens in the evolutionary phases of development (Cimino et al., 2012b), research has underlined that when it occurs in the first 3 years of life, it has a greater impact on psychological and physical health (Abdelnoor and Hollins, 2004). However, within the Developmental Psychopathology framework, many studies have evidenced that the early experience of parental death need not lead to the development of mental and physical illness, emphasizing individuals’ resilience capacities (Lin et al., 2004; Ahern et al., 2008) and suggesting the importance of identifying risk factors that can influence short- and long-term outcomes (Luecken and Roubinov, 2012; Williams and Aber, 2016).

In spite of these results, only a few studies have been interested in the possible mechanisms underlying the associations between early parental loss and later mental and physical health, focusing on the quality of emotional-social support following the death (Schoenfelder et al., 2011). The present longitudinal study aims to understand better the link between parental death in infancy and physical illness during adolescence, considering the role played by youths’ and surviving parents’ psychological profiles.

Within the psychodynamic perspective (Freud, 1917; Abraham, 1924; Brown, 1966), the childhood loss of a love object was postulated as an important risk factor for later psychopathology. The attachment theory proposed by Bowlby (1980), and the psychoanalytic position proposed by Freud (1917) in relation to the relationship between object loss and future depression, suggested that unresolved childhood grief might have both immediate and long-term effects, increasing vulnerability to future psychopathological problems (Bowlby, 1960, 1973). Since then, there has been increase in the empirical work focusing on the impact of parental death experiences on an individual’s psychological wellbeing, suggesting that the loss of an attachment figure might be associated with a higher risk of developing a wide range of later psychopathologies, such as panic disorder and phobia (Kendler et al., 1992), depression (Brown and Harris, 1978; Harris et al., 1986), and anxiety (Faravelli et al., 1985; Bifulco et al., 1987), especially when the death of a parent was experienced during an early stage of life. In this field, the pioneering studies by Spitz (1945) and Spitz and Wolf (1946) were among the first observations of the impact of early prolonged emotional deprivation on infants’ psychological and physical wellbeing, reporting the presence of both severe mental adverse outcomes (such as acute anxiety, distress, depression, and withdrawal) and a progressive physical deterioration, including frequent illnesses, motor delay, loss of weight, eczema, and, in some cases, even death. Furthermore, these studies suggested that parental loss in itself did not lead to later negative outcomes. Rather, other antecedent and concomitant risk factors associated with bereavement might influence physical and psychopathological health during the lifespan, including the quality of subsequent parental care (Breier et al., 1988) and a socio-economically disadvantaged status (Harris et al., 1986).

From a developmental point of view, as suggested by studies by Brent et al. (2012) and Feigelman et al. (2017), adolescence represents a particular risk stage for the onset of psychopathological symptoms among children who have experienced the early death of a parent (see also McLaughlin et al., 2012). During adolescence, a maturation of cognitive structures and functions occurs, leading to the acquisition of concepts such as the irreversibility and causality of events connected with the concept of death (Corr, 1995; Cerniglia et al., 2015) and involving a re-elaboration of past traumas, such as the death of a parent (Blos, 1962). In fact, empirical research has widely confirmed that childhood parental loss is a crucial risk factor for adolescent onset and maintenance of both internalized and externalized psychopathological difficulties (Kaplow et al., 2010; Stikkelbroek et al., 2012), such as depression (Melhem et al., 2008; Jacobs and Bovasso, 2009; Babore et al., 2016; Feigelman et al., 2017), suicidality (Serafini et al., 2015), and an increased tendency to risky behaviors, including substance abuse (von Sydow et al., 2002; Cerniglia et al., 2017b). Moreover, there is evidence that early parental death is significantly associated with eating disorder symptoms (Beam et al., 2004; Nickerson et al., 2013; Cerniglia et al., 2017a) and dissociative symptoms (Cerniglia et al., 2014), which may represent maladaptive strategies used by adolescents to cope with negative emotions related to loss (Cimino et al., 2012a).

In adolescence, the impact of early parental death also seems to affect other domains associated with the specific evolutionary tasks of this phase. In particular, adolescence is described as a transitional period of consolidation and integration of identity transformations (Blos, 1977). Studies have shown that in this developmental period, the early death of a parent is also associated with difficulties in interpersonal and intimate bonds (Maier and Lachman, 2000), lower grades or school dropout (Cas et al., 2013; Berg et al., 2014), and lower employment rates (Ellis et al., 2013). Furthermore, a growing body of research has evidenced that besides the impact of this early adverse experience on individual emotional-behavioral functioning, early parental death also plays a crucial predictive role for negative physical health outcomes in adolescence (McEwen and Lasley, 2002; Luecken, 2008; van de Pavert et al., 2017), such as chronic illness (Raposa et al., 2014), cortisol dysregulation (Biank and Werner-Lin, 2011), and premature death (Li et al., 2014; Smith et al., 2014). In particular, epidemiological studies on the effects of adverse childhood experiences (ACEs) on an individual’s health throughout the lifespan development have reported an increased risk of developing a wide spectrum of physical illnesses (Felitti et al., 1998; Felitti and Anda, 2003; Anda et al., 2006; Felitti, 2009). With specific regard to early parental loss, more contemporary ACE-related studies have shown that this experience was prospectively associated with adolescent obesity, unhealthy weight control behaviors (Isohookana et al., 2016), heart disease, asthma (Scott et al., 2011), and painful medical conditions, including arthritis or rheumatism, chronic back and neck problems, severe migraines or headaches, and other forms of chronic pain (Sachs-Ericsson et al., 2017). Overall, these findings are hypothesized to result from altered stress responses over a lifetime that could increase the risk for physical difficulties, including hypertension, asthma, and heart disease (Wright, 2011; Cassel, 2017). Raposa et al. (2014) found that early stress conditions are related to the presence of depression during adolescence, which could have a negative impact on physical health in early adulthood.

Recent studies have suggested that the main mechanisms responsible for increased vulnerability to psychological and physical illnesses after parental death in childhood arise from the impact of this event on the system of response to physiological stress (Pendry and Adam, 2007). In accordance with transitional events theory (Felner et al., 1988), variability in children’s adjustment after the loss of a parent is influenced by the chain of stressful life events that follow the death, children’s protective resources, and the dynamic relationships between these variables. More specifically, early parental death represents a stressful experience that may overwhelm a child’s ability to cope with this event (Goodman, 2002), initiating a cascade of negative life events that affect the development of emotional and self-regulatory abilities to respond to daily stressors over time (Luecken and Lemery, 2004; Tottenham et al., 2011). At a neurobiological level, it may induce important biological changes that impact nervous, endocrine, and immune systems (Danese and McEwen, 2012), which causes their effects to persist throughout the lifespan (Rutter, 1996; Adejuwon, 2010), increasing children’s vulnerability to both psychopathological and physical illness (Luecken et al., 2009; Flaherty et al., 2013; Serafini et al., 2015).

In this regard, among the primary resources that could help a child to adapt positively to his/her parental loss are the quality of the relationship with the surviving parent (Compas et al., 2001; Eisenberg et al., 2001) and the ability to cope with future stressful life events (Kwok et al., 2005; Haine et al., 2006; Pendry and Adam, 2007). Furthermore, the quality of the relationship with the surviving parent may be impaired by the maladaptive psychopathological functioning of the caregiver, representing a significant risk factor for the bereaved offspring’s later psychological and physical health (Wolchik et al., 2008). Several studies have shown that the presence of parental psychopathological symptoms is prospectively associated with the development of psychopathological difficulties in offspring over time (van der Pol et al., 2016; Hannigan et al., 2017; Plass-Christl et al., 2017). A parent with psychological problems may provide a family environment that does not promote children’s emotional regulation, leading children to develop ineffective emotional regulation strategies (Luecken et al., 2005), resulting in excessive emotional and physiological reactivity to later stress in adolescence (Luecken et al., 2009; Tafà et al., 2017).

On the basis of the present literature, the aims of this 6-year longitudinal study were to (1) assess the possible predictive values of offsprings’ and parents’ psychopathological symptoms for the occurrence of physical problems in adolescents and (2) determine the sex differences in the predictive value of psychopathological symptoms for the occurrence of physical problems in adolescents.

## Materials and Methods

### Participants

The subjects recruited for this study were part of a larger sample (*N* = 3548 children) and were recruited in the first grade of elementary schools in central Italy in March 2010. All students from four randomly selected classes in each school were invited to contribute to the study. The research group was composed of five psychologists who explained the goals and procedure of the study to the students and teachers in their classrooms and to their parents in two information sessions. In addition, the children’s pediatricians collaborated in the study, providing information on the state of the children’s physical health at different stages of the research.

In accordance with the Declaration of Helsinki, the study was approved before its start by the Ethical Committee of the Faculty of Psychology at Uninettuno University (n. 2/2010), and written informed consent was obtained from the parents before assessment.

From the larger sample, we selected for the aims of this study adolescents who had lost a parent in their first 3 years of life (*N* = 418 adolescents, 53% boys).

The assessments were carried out in four steps over 6 years. The mean (SD) age of the children at Time 1 was 6.31 (0.42) years; Time 2 was at 8 years of age; Time 3 was at 10 years of age; and Time 4 was at 12 years of age. The surviving parents’ mean age at Time 1 was 43.55 (2.3) years. At Time 2, the surviving parents’ mean age was 45.85 (1.9) years; at Time 3, it was 47.91 (2.4) years; and at Time 4, it was 50.01 (1.4) years. Sixty-two percent of surviving parents were mothers. All the children were of homogeneous nationality and were their parents’ biological children. Most families were of middle socioeconomic status (94%) (SES; Bornstein and Bradley, 2003).

### Measures

#### Physical Health

Physical health assessment was measured by pediatricians at all sessions through a modified questionnaire derived from Health Appraisal Questionnaire of the Centers for Disease Control and Prevention. The questions concerned gastrointestinal diseases (irritable bowel symptoms, duodenal, or gastric ulcer), cardiovascular diseases (hypertension, angina pectoris), and respiratory symptoms and diseases (asthma, chronic bronchitis, shortness of breath). A detailed description of this measure and its characteristics can be found in Felitti et al.’s (1998) work.

*Children’s psychopathological symptoms* were assessed at all session through the CBCL 6-18 (CBCL; Achenbach and Rescorla, 2001; Italian validation – Frigerio et al., 2004), which is a report-form measure filled out by parents (in this study, it was filled out by the surviving parent) that assesses children’s emotional-behavioral functioning. It contains 113 items that are rated as Not True (0), Somewhat or Sometimes True (1), or Very True or Often True (2). Validity and reliability are excellent, and extensive normative data are available for children ranging from 6 to 18 years (Frigerio et al., 2004). In this tool, Anxious/Depressed, Withdrawn/Depressed, and Somatic Complaints are grouped into the subscale of Internalizing Problems; Rule-Breaking Behavior and Aggressive Behavior are grouped into the subscale of Externalizing Problems; in addition, Social Problems, Thought Problems, and Attention Problems (not grouped into any subscale) are also considered. For the aims of this study, we used DSM-oriented scales (oriented to the DSM-IV-TR, since the study began before the publication of the fifth version): affective problems, anxiety problems, somatic problems, attention deficit/hyperactivity problems, oppositional defiant problems, and conduct problems. We used the clinical cut-offs for the DSM-oriented scales and thus regarded these variables as categorical.

#### Dissociation

The Child Dissociative Checklist (Putnam et al., 1993) is a 20-item observer-report checklist with a 3-point scale (0 = not true, 1 = sometimes true, 2 = frequently true). The Child Dissociative Checklist is a clinical screening instrument that assesses dissociation on the basis of ratings given by caregivers or adults in close contact with the child. A score of 12 or higher on the Child Dissociative Checklist is evidence of pathological dissociation. The Child Dissociative Checklist shows good 1-year test–retest stability (*r* = 0.65) and internal consistency (Cronbach’s alpha = 0.86) (Putnam et al., 1993). Good convergent and discriminant validity have been indicated (Putnam et al., 1993).

#### Parental Psychopathological Risk

The Symptom Check-List (SCL-90-R) is a 90-item self-report symptom inventory aimed at measuring psychological symptoms and psychological distress (Derogatis, 1994). Its main symptom dimensions are Somatization, Obsessive-Compulsivity, Interpersonal Sensitivity, Depression, Anxiety, Hostility, Phobic Anxiety, Paranoid Ideation, and Psychoticism. The SCL-90-R has been shown previously to have good internal coherence (α coefficient = 0.70–0.96) in adolescents and adults (Italian validated version: Prunas et al., 2012). Besides these nine primary scales, the questionnaire provides a Global Severity Index (GSI). The GSI is obtained from the average severity score of all the symptoms indicated by the subjects, making it possible to determine severity and degree of psychological distress. The clinical cut-off for this index is 1 (Prunas et al., 2012). In this study, we chose to use the GSI as a global index of psychopathology, following the suggestion of Bergly et al. (2013) who pointed out a high collinearity between the nine syndromic subscales.

### Procedure and Statistical Analysis

The parents of the recruited subjects completed the assessments for demographic information, offspring emotional-behavioral functioning, and their dissociation symptoms at their home, while the pediatricians filled out the measure to assess physical health at schools. Parents also filled out the measure for the screening of their own psychopathological risk (at home). Parents and pediatricians were asked to fill out the measures at 6, 8, 10, and 12 years of the child. To determine the risk factors for physical problems, data from participants who were classified as healthy in the initial assessment were selected for statistical analysis. Statistical analyses were performed using the Statistical Package for the Social Sciences, SPSS software (IBM corp, 2013). A univariate Cox proportional hazard regression analysis with time-dependent variables was used to examine the predictive values of baseline psychopathological symptoms (assessed through the CBCL/6-18 and SCL-90-R), dissociation, and demographic characteristics (sex and age) for the occurrence of physical problems during the 6-year period of follow-up. The formula of the Hazard Function was H(*t*) = H_0_(*t*) x exp(b_1_X_1_ + b_2_X_2_ + b_3_X_3_ +... b_k_X_k_, where X_1_... X_k_ are a collection of predictor variables and H_0_(*t*) is the baseline hazard at time *t*, representing the hazard for a person with the value 0 for all the predictor variables.

The outcome variable (survival time) in the Cox proportional hazard regression was defined as the period between the initial assessment and the detection of occurrence of physical problems during follow-up. If a participant showed a relevant physical problem in a follow-up assessment, an event was recorded and that case’s data were not censored. On the other hand, if no relevant physical problem was detected by the pediatrician in any follow-up assessment, no event was recorded and the participant’s data were censored. Thus, censored individuals were those who either had no physical problem by the end of the study or were lost to follow-up during the course of the study before the physical problem had been identified. We examined the predictive values of sex and age for the occurrence of physical problems using the univariate Cox proportional hazard regression, and then we evaluated the predictive values of the psychopathological symptoms on the basis of those indicated by Peduzzi et al. (1995). All significant psychopathological predictors found in the univariate analysis were then further used in a forward stepwise multivariate Cox proportional hazard regression to determine which psychopathological symptom was the most significant predictor of physical problems among all, male, and female participants. A *p*-value lower than 0.05 was considered significant.

## Results

A total of 418 mourning families completed the protocol, and all children were assessed by pediatricians in the course of their development toward adolescence. Of these, 12% (*N* = 50) were classified as having a physical problem at Time 1 (52% females). Three-hundred and sixty-eight children (51% females) showed no relevant physical problem at the first assessment at 6 years of age.

To examine the predictive values of offsprings’ and parents’ psychopathological symptoms and offsprings’ dissociation and demographic characteristics (sex and age) for the occurrence of physical problems during the 6-year period, we carried out a univariate Cox proportional hazard regression analysis with time-dependent variables. In particular, we examined the predictive values of sex and age for the occurrence of physical problems using a univariate Cox proportional hazard regression and then we evaluated the predictive values of the psychopathological symptoms.

The results of the univariate Cox proportional hazard regression analysis are shown in **Tables 1**, **2** and indicate that, irrespective to sex, offsprings’ affective problems and dissociation and surviving parents’ GSI were risk factors for the occurrence of a relevant physical problem (**Table 1**).

**Table 1A:** Predictive value of sex and psychopathological problems for the occurrence of a relevant physical problem in the univariate Cox proportional hazard regression.

Variable (no. missing data)	Number of relevant physical problems		Wald χ^2^ (*p*-values)	Hazard ratio (95% confidence interval)
	Event	Censored		
Sex				2.25 (1.46–2.69)
M	87	135	3.12 (*p* = 0.063)	1
F	91	105		
Affective problems (2)			22.42^∗^ (*p* = 0.033)	1.96 (1.36–2.41)
Yes	47	176		1
No	75	118		
Anxiety problems (4)			19.71^∗^ (*p* = 0.024)	2.12 (1.67–2.41)
Yes	52	98		1
No	162	102		
Somatic problems (1)			4.11 (*p* = 0.071)	1.23 (1.12–2.25)
Yes	27	129		1
No	133	128		
Attention Deficit/Hyperactivity problems (2)			5.21 (*p* = 0.083)	2.24 (1.32–2.68)
Yes	19	149		1
No	108	140		
Oppositional Defiant problems (0)			4.12 (*p* = 0.085)	2.51 (1.44–2.99)
Yes	22	164		1
No	105	127		
Conduct problems (3)			3.51 (*p* = 0.091)	2.31 (1.41–2.72)
Yes	47	105		1
No	145	118		
Dissociation (5)			15.51^∗^ (*p* = 0.021)	2.14 (1.38–2.62)
Yes	51	153		1
No	111	98		
GSI (2)			12.49^∗^ (*p* = 0.013)	2.19 (1.36–2.71)
Yes	46	128		1
No	143	99		

*Missing data were unavailable due to incomplete responses from surviving parents for the administered measures or missing responses from the pediatricians about the physical health of the children. No, Number; GSI, Global Severity Index; ^∗^p < 0.05; ^∗∗^p < 0.01; ^∗∗∗^p < 0.001.*

Moreover, as can be seen in the procedure section, the children’s and parents’ psychopathological symptoms were input into a forward stepwise multivariate Cox proportional hazard regression (see **Table 2**) to establish which symptomatic manifestation showed the highest predictive value for the occurrence of a physical problem. The results indicated that offspring dissociation was the most significant predictor for the occurrence of relevant physical problems after controlling for sex and age. The most prevalent physical problem was respiratory symptoms (54%), followed by gastrointestinal diseases (35%) and cardiovascular diseases (11%).

**Table 1B:** Number of cases with relevant physical problems at each follow-up assessment point.

	Time 2	Time 3	Time 4
M 87	31	34	22
F 91	28	36	27

**Table 2:** Predictive value of age, sex, and psychopathological symptoms for the occurrence of a relevant physical problem: Multivariate Cox proportional hazard (forward) regression.

Variable (no. missing data)	Wald χ^2^ (*p*-values)	Hazard ratio (95% confidence interval)
All participants (21)		
Age	0.04 (*p* = 0.67)	0.89 (0.76–1.37)
Sex	0.07 (*p* = 0.81)	0.99 (0.79–1.42)
Affective problems	9.92^∗^ (*p* = 0.028)	1.65 (1.21–2.12)
Anxiety problems	11.73^∗∗^ (*p* = 0.008)	2.23 (1.42–2.52)
Dissociation	16.72^∗∗∗^ (*p* = 0.0009)	2.32 (1.48–2.84)
GSI	14.22^∗^ (*p* = 0.026)	1.59 (1.11–2.02)

*Missing data were unavailable due to incomplete responses from surviving parents for the administered measures or to missing responses from the pediatricians about the physical health of the children. No, Number; GSI, Global Severity Index. ^∗^p < 0.05; ^∗∗^p < 0.01; ^∗∗∗^p < 0.001*.

## Discussion

The loss of a parent in childhood is one of the most significant painful events for a child’s emotional development. Several studies have highlighted that individuals who have lost a parent in early childhood have a greater risk of developing depressive symptoms in adolescence (Melhem et al., 2007, 2008) and that this loss also affects physical health (Abdelnoor and Hollins, 2004). However, there are few studies interested in the possible mechanisms underlying the associations between parental loss and subsequent mental and physical health (Luecken et al., 2009; Sachs-Ericsson et al., 2017) or evidencing the central role of ongoing stress exposure (Raposa et al., 2014). The current longitudinal study aims to understand better the link between parental death during childhood and physical illness during adolescence, considering the role played by the psychological profiles of surviving parents and offspring. In particular, this study aims to assess the possible predictive values of children’s and parental psychopathological symptoms for the occurrence of physical problems in adolescents.

To examine the predictive values of offspring’s and parents’ psychopathological symptoms, dissociation, and demographic characteristics (sex and age) for the occurrence of physical problems during a 6-year period, we carried out a univariate Cox proportional hazard regression analysis with time-dependent variables. Results showed that, regardless of sex, offspring’s affective problems and dissociation and parents’ GSI were risk factors for the occurrence of relevant physical problems.

With regard to the surviving parent’s psychopathological risk, the international literature has underlined that a relationship characterized by warmth, emotional support, and acceptance (i.e., positive or effective parenting) is associated with lower psychopathological difficulties in bereaved children and adolescents (Hagan et al., 2011, 2012). By contrast, a surviving parent who is struggling emotionally herself/himself over the loss of his/her partner may be less sensitive and supportive to grief-related emotions in their children (Werner-Lin and Biank, 2013). The ways in which the surviving parent faces the loss of their own partner influence how their children handle mourning (Kirwin and Hamrin, 2005) and their adaptation to life (Saldinger et al., 2004). Furthermore, a parent with a psychological problem may provide a family environment that does not promote their children’s emotional regulation, leading children to develop ineffective emotional regulation strategies (Luecken et al., 2005), resulting in excessive emotional and physiological reactivity to later stress in adolescence (Luecken et al., 2009). In addiction, interestingly, several studies have found that parental roles and couple dynamics are associated with children’s healthy behaviors and adherence to medical regimes, which are critical elements of cardiovascular rehabilitation (Martire and Helgeson, 2017; Smith and Baucom, 2017).

Our findings would seem to confirm the influence of parental psychological difficulties and the emotional problems of children on their physical health. In order to understand the predictive value of these variables better and to determine which psychopathological symptom was the most significant predictor of physical problems, we used all significant psychopathological predictors found in the univariate analysis in a forward stepwise multivariate Cox proportional hazard regression. Results showed that offspring dissociation was the most significant predictor for the occurrence of relevant physical problems after controlling for sex and age.

According to our study, both the psychopathological parental risk and the youth dissociation contribute to physical problems, although the main predictor of these is dissociation. We can assume that the traumaticity of the event involves not only the loss of the parent but also the consequent change in the relationship with the surviving parent. In addition, Schimmenti and Caretti (2016) have suggested that dissociation is a key variable in understanding clinical disorders that have their roots in relationally traumatic experiences during childhood. The authors, focusing on emotionally traumatic experiences during childhood, emphasized how dissociation could, paradoxically, protect the child through multiple disconnections in the self, which occur at both a mental and bodily level. Furthermore, Schimmenti (2017) has argued that excessive activation of dissociative processes following interpersonal trauma during childhood can hinder cognitive and affective treatment of information and the integration of mental, behavioral, and somatic states, including at the neurobiological level (Frewen et al., 2008). This may result in an increased risk of emotional dysregulation and exposure to further traumatic experiences, resulting in maladaptive developments. Putnam (1997) stressed how these alterations in consciousness reflect an inability to integrate or combine information and experiences in a normally predictable way. Therefore, thoughts and emotions are disconnected, somatic sensations are outside conscious awareness, and behavioral repetitions occur without deliberate choice, planning, or self-consciousness (Cook et al., 2017).

It is important to note that, in our sample, it appears that dissociating symptoms predict physical diseases in the specific period of puberty. Adolescence makes its debut with puberty. In this period, there are two changes that are fundamental to the psychophysical development of the individual: pubertal development and the consolidation of logical-formal thinking that makes it possible to organize the contents of the experience. Boys and girls go through a rapid and often unexpected bodily maturation, with the consequent modification of the relationship with themselves that is often perceived as outside their control. In addition, during adolescence, the maturation of cognitive structures and functions allows the acquisition of concepts, such as irreversibility and causality of events, connected with the concept of death (Corr, 1995), allowing a re-elaboration and control of childhood traumas.

On the basis of our results and Bucci’s (2003) multiple code theory, we hypothesized that the dissociative defense used by a child during childhood manifests itself in adolescence with a greater difficulty in understanding her/his emotions, which can manifest itself in physical symptoms. In this respect, further research should also take into account the alexithymic difficulties in adolescents who have lost a parent in childhood.

The present study has some limitations. First, the gender of the missing parent was not considered. Weller et al. (1991) found that children who lost their father figure had more symptoms of depression than did children who lost their mother. However, the scientific literature contains conflicting data. Some studies found that higher adolescent psychopathological risk was associated with the early death of the mother rather than the father (Brown et al., 1977; Kunugi et al., 1995), while other studies highlighted that losing the same-sex parent in childhood had a greater negative impact (Takeuchi et al., 2003). These conflicting results may be linked to the second limitation of this study: The cause of parent’s death was not considered, and it is possible that different causes of death lead to very different living environments for the child. As Dowdney (2000) points out, it is important to investigate how the parent’s death occurred, such as whether there was murder, suicide, or death after a long illness. Third, our research did not take into account the social network that could have been of support to adolescents during their development; a good relationship with the extended family and/or peers could be important protection factors.

On the other hand, this study has several strengths. In particular, this is a longitudinal study, which takes into consideration a large sample of children who have lost a parent in early childhood, following them with assessments until early adolescence. Numerous studies have in fact underlined the importance of this delicate passage (Blos, 1977), with the consequent modifications linked to puberty. Furthermore, in these assessments, we evaluated both the psychopathological symptoms of parents and children, as well as the offsprings’ physical symptoms, taking into consideration various channels of symptomatic manifestation, in a period of development in which the body becomes a central element in the manifestation of individual suffering. These results can be relevant and add to the previous literature in the field of prevention and intervention practices in samples of adolescents who lost a parent in their early childhood, orientating clinical work.

## Author Contributions

MT designed the study and wrote the draft of the introduction section. LC wrote the introduction and the methods sections. SC wrote the discussion section and the draft of the introduction section. GB and EM performed statistical analyses. RT supervised the study and approved the final draft.

## Conflict of Interest Statement

The authors declare that the research was conducted in the absence of any commercial or financial relationships that could be construed as a potential conflict of interest.
